# TRIDENT-Risk: An AI-driven temporal ensemble framework for proactive high-severity accident risk forecasting in Intelligent Transportation Systems

**DOI:** 10.1371/journal.pone.0357089

**Published:** 2026-10-07

**Authors:** Sanchit Vashisht, Shalli Rani, Fazlullah Khan

**Affiliations:** 1 Chitkara University Institute of Engineering and Technology, Chitkara University, Rajpura, Punjab, India; 2 School of Computer Science, Faculty of Science and Engineering, University of Nottingham, Ningbo China, Ningbo, Zhejiang, China; Nanjing Forestry University, CHINA

## Abstract

High-severity traffic accidents impose substantial human and economic burdens, necessitating proactive and data-driven risk forecasting mechanisms within intelligent transportation systems. This study proposed TRIDENT-Risk, a temporal risk-driven imbalance-optimized ensemble framework for high-severity accident prediction using the eXtreme Gradient Boosting (XGBoost) model. The approach integrates spatial–temporal aggregation, multi-scale lag and rolling risk features, imbalance-aware gradient boosting, and threshold optimization to enhance minority-class detection. Experimental evaluation using publicly accessible US-Accidents dataset including about 7.7 million accident records from February 2016 to March 2023 demonstrated that the proposed TRIDENT-Risk achieved a ROC-AUC of 0.8917, PR-AUC of 0.7266, precision of 0.60, recall of 0.81, F1-score of 0.69 and accuracy of 0.82. Compared with the strongest baseline (MLP), the proposed framework improved high-risk recall from 0.70 to 0.81 (15.7% relative improvement), increased ROC-AUC from 0.8776 to 0.8917 and improved PR-AUC from 0.7016 to 0.7266, demonstrating superior minority-class detection and reliable proactive accident forecasting. Temporal grid-based aggregation was used to frame high-severity accidents as a binary prediction problem and an 80:20 chronological train-test split was used to eliminate temporal leaking. The proposed framework offers a scalable and interpretable solution for proactive traffic safety management and supports intelligent decision-making in next-generation transportation systems.

## 1. Introduction

Road transportation systems form the backbone of modern economies, enabling mobility, trade, and social connectivity at an unprecedented scale [1,2]. However, the rapid expansion of vehicular networks has been accompanied by a persistent rise in traffic congestion, infrastructure strain, and, most critically, road traffic accidents [3,4]. High-severity accidents, in particular, impose substantial human, social, and economic costs, including loss of life, long-term injury, medical expenditure, traffic disruption, and infrastructure damage [5,6]. According to global transportation safety reports, road crashes remain one of the leading causes of death worldwide, highlighting the urgent need for intelligent and proactive safety management mechanisms within transportation ecosystems. The evolution of intelligent transportation systems (ITS) has introduced advanced sensing, data collection, and communication capabilities into traffic infrastructure [7]. Large-scale accident repositories, weather records, and temporal traffic logs now provide a rich foundation for predictive analytics [8,9]. These developments have enabled a transition from reactive accident response strategies toward predictive and preventive safety management [10,11]. Instead of merely responding to incidents after occurrence, modern transportation analytics aims to anticipate high-risk conditions and support early intervention through data-driven decision systems. In this context, forecasting high-severity accident risk at regional and temporal scales has emerged as a critical research challenge [12,13].

Accident risk prediction is inherently complex. Traffic incidents are influenced by dynamic interactions among environmental conditions, human behavior, infrastructure characteristics, and temporal patterns [14,15]. Weather fluctuations, visibility conditions, humidity levels, historical accident recurrence, and time-of-day variations collectively shape the probability of severe events. Furthermore, accident datasets typically exhibit strong class imbalance, as high-severity crashes occur less frequently than minor incidents, creating additional modeling challenges [16]. Conventional statistical approaches often struggle to capture nonlinear temporal dependencies and imbalanced distributions effectively, while purely deep learning (DL) based spatial architectures may introduce unnecessary complexity when the dominant predictive signals are temporal and contextual rather than strongly graph-structured [17,18]. Recent advancements in machine learning (ML), particularly ensemble-based gradient boosting techniques, have demonstrated strong performance in tabular and structured data environments [19,20]. These models effectively capture nonlinear interactions, adapt to heterogeneous feature distributions, and offer inherent robustness against overfitting [21,22]. Nevertheless, direct application of generic boosting models may not sufficiently address temporal dependencies across multiple horizons, risk accumulation effects, or imbalance-sensitive optimization objectives. In safety-critical domains, detection sensitivity for high-risk regions is often more important than maximizing overall accuracy, necessitating risk-aware threshold calibration and imbalance-adjusted training strategies [23].

To address these challenges, a temporal risk-driven ensemble modeling paradigm is proposed for high-severity traffic accident forecasting. The framework integrates multi-scale temporal feature engineering, imbalance-aware optimization, and decision-threshold calibration within a unified boosting architecture. Multi-horizon lag variables, rolling statistics, and risk acceleration indicators are incorporated to capture short-term and medium-term temporal dynamics. Class imbalance is explicitly handled through weighted optimization to enhance sensitivity toward high-risk events. Furthermore, threshold tuning is employed to balance precision–recall tradeoffs in accordance with safety-oriented operational priorities. This integrated design aims to maximize early detection capability while maintaining stable discriminative performance. Extensive experimentation is conducted using large-scale accident and contextual datasets aggregated at regional grid levels. The proposed framework is systematically benchmarked against widely adopted baseline models, including logistic regression, random forests, gradient boosting, and multilayer perceptrons. Comparative evaluation is performed using multiple metrics, including ROC-AUC, PR-AUC, recall for high-risk events, and F1-score, ensuring a comprehensive assessment under imbalanced conditions. Empirical results demonstrate that the proposed temporal risk-driven ensemble model achieves superior discriminative capability and significantly improved high-risk recall compared to baseline approaches, thereby reinforcing its suitability for proactive safety management within intelligent transportation environments.

### 1.1. Contribution

The contributions of this study are as follows:

- Proposes TRIDENT-Risk, a temporal risk-driven ensemble framework specifically designed for high-severity accident forecasting.
- Introduces multi-scale temporal risk descriptors including lag features, rolling statistics and risk acceleration indicators.
- Incorporates imbalance-aware gradient boosting together with adaptive threshold optimization for improving minority-class detection.
- Performs extensive evaluation on a large-scale US accident dataset and demonstrates superior ROC-AUC, PR-AUC and recall compared with four representative ML baselines.
- Provides interpretable accident-risk analysis through SHAP explanations and spatial hotspot validation.

### 1.2. Structure

The rest of this paper is organized as follows. Section 2 reviews the existing literature of traffic accident prediction and identifies the research needs that underlie the proposed methodology. Section 3 provides theoretical preliminaries, problem formulation, evaluation criteria and baseline models for comparison analysis. Section 4 presents the proposed TRIDENT-Risk framework encompassing spatial-temporal aggregation, multi-scale temporal feature engineering, imbalance-aware gradient boosting and risk-sensitive threshold optimization. Section 5 explains the experimental setup, dataset description, preprocessing steps, hyperparameter setting and evaluation protocol. In Section 6, the experimental results, the performance comparison, the interpretability analysis and the discussion of the results are presented. Section 7 presents limitations of the proposed framework and some avenues for future research. The paper concludes with Section 8, which summarizes the main findings and contributions.

## 2. Literature work

Li et al. [24] examined the transportation safety risk linked to extremely volatile lithium-ion batteries utilized in electric vehicles and other devices. The Bayesian framework used historical accident data and expert knowledge to find at how likely fire-related mishaps are over time. Results showed that the risk of transport goes up with time; after four hours, the severity of accidents goes up by 12.28% compared to the first stage. The most important causes of fatal accidents are battery self-heating (92.2%) and inappropriate handling (72.4%). Rashid et al. [25] addressed the increasing global issue of car accidents by suggesting a complete explainable artificial intelligence (XAI) framework to help different groups of people make decisions based on evidence. It presented a systems-level taxonomy that classifies driver-related behaviors and other relevant elements linked to different levels of harm severity. In the second phase, they built and improved several predictive models, such as XGBoost, RF and DNN. Then, model-agnostic SHAP explanations were employed to make sense of the predictions. Fan et al. [5] examined the crucial issue of human weariness in maritime transport, where diminished seafarer performance markedly elevates the likelihood of accidents and undermines safety at sea. Methodologically, it formulated a methodology for identifying important Risk Influencing Factors (RIFs) that contribute to human weariness, utilizing historical accident and event data. It combines the Least Absolute Shrinkage and Selection Operator (LASSO) with Bayesian Networks (BN) to make a logical and understandable forecasting model. Adewopo et al. [26] provided a thorough examination of accident detection methodologies designed to enhance road safety and traffic management in smart cities. To overcome the shortcomings of current methods, a new lightweight architecture called I3D-CONVLSTM2D suggested for smart city surveillance systems to detect accidents in real time. The model combines RGB frames with optical flow data to effectively capture changes in both space and time. The I3D-CONVLSTM2D RGB + Optical-Flow (trainable) setup gets 87% Mean Average Precision (mAP), which is better than other setups. Sun et al. [27] proposed a highway collision risk assessment framework combining matched case-control analysis, multi-parameter traffic state clustering and Bayesian conditional logistic regression was suggested to quantify the crash risk under diverse traffic situations. The results demonstrated that different traffic safety statuses are associated with different crash risk levels, giving a basis for proactive highway traffic safety management. Li et al. [28] developed a correlated random parameters accelerated failure time model with variability in means and variances to analyze factors influencing highway traffic conflict interval times. The system utilizes the trajectory data of the HighD dataset to capture heterogeneity and interaction effects across traffic variables, showing better performance and delivering useful insights for enhancing road safety and traffic management decisions. Huang et al. [29] created a diagnostic framework of Geographically Weighted Regression and Random Forest interpretation to uncover spatiotemporal mismatches of traffic collision risks and safety governance. Risk migration to suburban regions, dominant behavioral risk factors and governance weaknesses that require dynamic, precision-based safety management based on 10 years of urban crash data. Hao et al. [30] presented a knowledge graph-based safety risk evaluation methodology to quantify the influence of unsafe driving behaviors on road transport safety. The approach integrates multisource accident data, CRITIC weighting, similarity analysis, and graph-based reasoning to effectively identify risky driving and driver competency as prominent risk variables and to trace causal routes between actions and accidents. Li et al. [31] examined the influencing elements of e-bike riders’ hit-and-run behavior based on 6022 crash records by employing a Light Gradient Boosting Machine and SHAP analysis. The findings point to various urban and rural risk configurations, highlighting the statistical relevance of the time, roadway, injury severity and violation-linked elements associated with hit-and-run crashes involving e-bike users. Megnidio et al. [7] investigated the enduring issue of road traffic deaths, which continue to be a predominant cause of death globally, notwithstanding numerous prevention strategies. The study employs three ML classification algorithms DT, LightGBM, and XGBoost to scrutinies UK road traffic accident data from 2020, utilizing both default and hyper-tuned parameters. Feng et al. [32] presented a data-driven risk analysis approach to tackle the recurrent incidence of marine accidents and the intricate interconnections among RIFs. The method combines association rule mining (ARM) with the fast FP Growth algorithm and complex network (CN) analysis to find common risk patterns and look at how the different elements are related to each other. Complex network theory is used to look at network features, find important RIFs using the weighted LeaderRank (WLR) technique, and do robustness analysis to see how stable the system is. Berhanu et al. [33] adressed the increasing issues of road traffic accidents (RTAs) and congestion in urban areas by suggesting a comprehensive framework for recommending safe routes. The RF model used data from accidents that happened between 2014 and 2019 to forecast crashes with 78% accuracy. Spatial network analysis used segments with lower expected crash counts and lower accident rates in the past to find safer options. The data showed that there were two routes: one that was 32.27 km long and took 50.78 minutes to travel, and another that was 28.6 km long and took 41.58 minutes. Shafique et al. [34] examined the prediction of accident severity in autonomous vehicles (AVs) to improve system reliability and foster public trust. To tackle the issue of data imbalance in the limited dataset of 334 samples, a new method called the Multi-Distance Synthetic Technique (MDST) was created to make the analysis more reliable. They used natural language processing techniques and ML models together to get useful information from written accident reports. Additionally, Recursive Feature Selection (RFS) was utilized to ascertain the most significant aspects for predicting severity. An ensemble learning model was trained using the optimized feature set, and it got a high accuracy of 0.92. The results show that using NLP, data balancing, and ensemble learning together works well for predicting the severity of AV accidents. A comprehensive comparison of existing studies with the proposed framework is presented in Table 1, highlighting key methodological differences, research gaps, and corresponding improvements.

**Table 1:** Comparison of existing literature with the proposed TRIDENT-Risk framework.

Study	Method	Contribution	Gap & TRIDENT-Risk Advantage
Li et al. [24]	Bayesian Network	Battery risk modeling	Domain-specific; TRIDENT-Risk enables large-scale traffic prediction
Rashid et al. [25]	XAI + CatBoost	Explainable severity prediction	No temporal modeling; TRIDENT-Risk adds temporal features
Fan et al. [5]	LASSO + BN	Risk factor analysis	Not predictive; TRIDENT-Risk performs forecasting
Adewopo et al. [26]	I3D + ConvLSTM	Vision-based detection	High computation; TRIDENT-Risk is lightweight
Megnidio et al. [7]	DT, XGBoost	ML severity prediction	Limited temporal features; TRIDENT-Risk adds lag/rolling
Feng et al. [32]	ARM + Network	Risk analysis	Descriptive; TRIDENT-Risk is predictive
Berhanu et al. [33]	Random Forest	Safe route prediction	Moderate accuracy; TRIDENT-Risk improves performance
Shafique et al. [34]	NLP + Ensemble	Text-based prediction	Small dataset; TRIDENT-Risk uses large-scale data
**Proposed**	Temporal Boosting	Risk-aware forecasting	Handles temporal dynamics, imbalance, and scalability

Table 1 highlights that existing studies predominantly focus on domain-specific risk analysis, explainability, or static machine learning approaches. Many approaches lack integrated temporal modeling, scalability, or effective handling of class imbalance, which are critical for real-world accident forecasting. The proposed TRIDENT-Risk framework addresses these limitations by combining multi-scale temporal feature engineering, imbalance-aware gradient boosting, and risk-sensitive threshold optimization. This results in improved predictive performance, enhanced high-risk detection capability, and better alignment with intelligent transportation system requirements.

### 2.1. Research gaps

Existing studies have greatly improved the prediction of traffic accidents with ML and DL methods. However, there are still some research gaps. First, many approaches only optimize the total accuracy while ignoring the identification of the minority class, which is more important in safety-critical contexts. Second, modeling temporal dependencies is usually done using either short-term lag features or sequential DL architectures, but relatively few research combine multi-scale temporal accumulation with imbalance-aware optimization. Third, threshold calibration is rarely implemented while it is important to trade off false alarms and missed detections. Also, many existing methodologies are limited in their interpretability which hinders their practical applicability for transportation authorities. These research gaps motivate the development of the proposed TRIDENT-Risk framework.

## 3. Preliminaries

This section introduces the theoretical foundations necessary for high-severity accident risk forecasting and formally presents the baseline models used for comparative evaluation. The discussion begins with the problem formulation and evaluation framework, followed by concise mathematical descriptions of the baseline classifiers.

### 3.1. Problem formulation

Let the transportation region be partitioned into spatial grids indexed by g∈{1,2,...,G} and discretized in time into hourly intervals indexed by t∈{1,2,...,T}. For each grid–time pair (*g*,*t*), a feature vector ${𝐱}_{g,t}\in {\mathbb{R}}^{d}$ is constructed, containing contextual and *t*emporal variables such as accident counts, weather attributes, and lag-based risk indicators. The target variable $y_{g,t}\in \{0,1\}$ denotes the occurrence of at least one hi*g*h-severity accident within a predefined future horizon.

The objective is to learn a mapping given by Eq. 1:


$$\begin{array}{c} f:{\mathbb{R}}^{d}\to [0,1] \end{array}$$ (1)

that estimates the conditional probability shown by Eq. 2:


$$\begin{array}{c} {\hat{p}}_{g,t}=P(y_{g,t}=1|{𝐱}_{g,t}) \end{array}$$ (2)

which represents the predicted high-severity accident risk for a given spatial–temporal unit. The prediction problem is characterized by class imbalance, where high-severity events occur less frequently than non-severe cases. Consequently, performance evaluation must consider discrimination capability and minority-class sensitivity rather than overall accuracy alone.

### 3.2. Evaluation metrics

To assess predictive performance comprehensively, multiple metrics are employed.

The Receiver Operating Characteristic Area Under Curve (ROC-AUC) measures the ability of the model to distinguish between positive and negative classes across all classification thresholds. Formally, it evaluates the tradeoff between true positive rate (TPR) and false positive rate (FPR).

Given a threshold τ, predictions are defined by Eq. 3:


$$\begin{array}{c} {\hat{y}}_{g,t}=\left\{ \begin{array}{cc} 1, & \text{if }{\hat{p}}_{g,t}\geq \tau ,\\ 0, & \text{otherwise}. \end{array}\right. \end{array}$$ (3)

Precision and recall for the high-risk class are defined by Eq. 4:


$$\begin{array}{c} \text{Precision}=\frac{TP}{TP+FP},\;\text{Recall}=\frac{TP}{TP+FN}, \end{array}$$ (4)

where *TP*, *FP*, and *FN* denote true positives, false positives, and false negatives, respectively.

The F1-score, which balances precision and recall, is given by Eq. 5:


$$\begin{array}{c} F1=\frac{2\cdot \text{Precision}\cdot \text{Recall}}{\text{Precision}+\text{Recall}}. \end{array}$$ (5)

The Precision–Recall Area Under Curve (PR-AUC) provides additional insight into performance under class imbalance and is particularly informative in safety-critical settings.

### 3.3. Baseline models

To ensure rigorous benchmarking, several widely adopted classification models are considered as baselines. These models represent linear, tree-based, ensemble-based, and neural network paradigms.

#### 3.3.1. Logistic regression.

Logistic regression models the log-odds of the positive class as a linear function of input features. The probability estimate is defined by Eq. 6:


$$\begin{array}{c} {\hat{p}}_{g,t}=\sigma ({𝐰}^{⊤}{𝐱}_{g,t}+b) \end{array}$$ (6)

where 𝐰 is the weight vector, *b* is the bias term, and σ(·) denotes the sigmoid function represented by Eq. 7:


$$\begin{array}{c} \sigma (z)=\frac{1}{1+e^{-z}}. \end{array}$$ (7)

Model parameters are learned by minimizing the binary cross-entropy loss given by 8:


$$\begin{array}{c} ℒ=-\sum_{g,t}\left[y_{g,t}\log({\hat{p}}_{g,t})+(1-y_{g,t})\log(1-{\hat{p}}_{g,t})\right]. \end{array}$$ (8)

Logistic regression provides a strong linear baseline and serves as a reference for evaluating nonlinear model improvements.

#### 3.3.2. Random forest.

Random Forest is an ensemble of decision trees constructed using bootstrap aggregation. Each tree is trained on a random subset of data and features, thereby reducing variance and improving generalization.

Given *M* trees $\{T_{m}{\}}_{m=1}^{M}$, the predicted probability is obtained by averaging represented by Eq. 9:


$$\begin{array}{c} {\hat{p}}_{g,t}=\frac{1}{M}\sum_{m=1}^{M}T_{m}({𝐱}_{g,t}). \end{array}$$ (9)

Each tree partitions the feature space into regions through recursive binary splits that minimize impurity measures such as the Gini index given by 10:


$$\begin{array}{c} G=1-\sum_{k=1}^{K}p_{k}^{2} \end{array}$$ (10)

where $p_{k}$ represents the proportion of samples belonging to class *k* in a node.

Random Forest effectively captures nonlinear relationships and interactions without requiring extensive feature preprocessing.

#### 3.3.3. Gradient boosting (XGBoost).

Gradient boosting constructs an additive model by sequentially fitting weak learners to the residual errors of previous iterations. The model at iteration *m* is expressed as Eq. 11:


$$\begin{array}{c} {\hat{y}}^{\left(m\right)}=\sum_{k=1}^{m}f_{k}(𝐱) \end{array}$$ (11)

where each $f_{k}$ is a regression tree.

The objective function consists of a differentiable loss component and a regularization term given by 12:


$$\begin{array}{c} ℒ=\sum_{g,t}\ell (y_{g,t},{\hat{y}}_{g,t})+\sum_{k=1}^{m}\Omega (f_{k}), \end{array}$$ (12)

where $\Omega (f_{k})$ penalizes model complexity.

XGBoost enhances classical gradient boosting by incorporating second-order optimization, regularization, and parallelized computation, leading to improved performance and stability in structured data environments.

#### 3.3.4. Multilayer perceptron (MLP).

The multilayer perceptron is a feedforward neural network consisting of fully connected layers with nonlinear activation functions. For a network with *L* layers, the forward propagation is defined as Eq. 13:


$$\begin{array}{c} {𝐡}^{\left(l\right)}=\phi \left({𝐖}^{\left(l\right)}{𝐡}^{\left(l-1\right)}+{𝐛}^{\left(l\right)}\right), \end{array}$$ (13)

where ${𝐡}^{\left(0\right)}={𝐱}_{g,t}$, ϕ(·) denotes a nonlinear activation function such as ReLU, and ${𝐖}^{\left(l\right)}$ and ${𝐛}^{\left(l\right)}$ are learnable parameters.

The final output layer produces a probability estimate via a sigmoid activation. Parameters are optimized using backpropagation and stochastic gradient descent to minimize cross-entropy loss. MLP models can capture complex nonlinear feature interactions but may require careful regularization and hyperparameter tuning to prevent overfitting.

### 3.4. Discussion

The selected baseline models encompass diverse modeling philosophies, ranging from linear probabilistic classifiers to tree-based ensembles and neural architectures. This diversity ensures that the proposed framework is evaluated against strong and representative alternatives. By systematically comparing performance across these baselines, the effectiveness of the proposed temporal risk-driven ensemble approach can be rigorously validated in subsequent sections. The selected grid resolution is a compromise between local spatial representation and sufficient sample density, thus alleviating the effect of sparsity while retaining computational efficiency.

## 4. Proposed methodology

This section presents the proposed TRIDENT-Risk framework, a temporal risk-driven imbalance-optimized ensemble architecture designed for high-severity accident forecasting. The methodology integrates large-scale accident data processing, multi-scale temporal feature engineering, imbalance-aware gradient boosting, and risk-sensitive threshold optimization into a unified predictive pipeline. The overall workflow of the proposed TRIDENT-Risk framework is illustrated in Fig 1. The architecture integrates spatial–temporal aggregation, multi-scale temporal feature engineering, imbalance-aware gradient boosting, and risk-sensitive threshold optimization within a unified predictive pipeline. The modular design ensures structured data transformation, robust minority-class learning, and interpretable risk forecasting for intelligent transportation systems.

**Fig 1:**
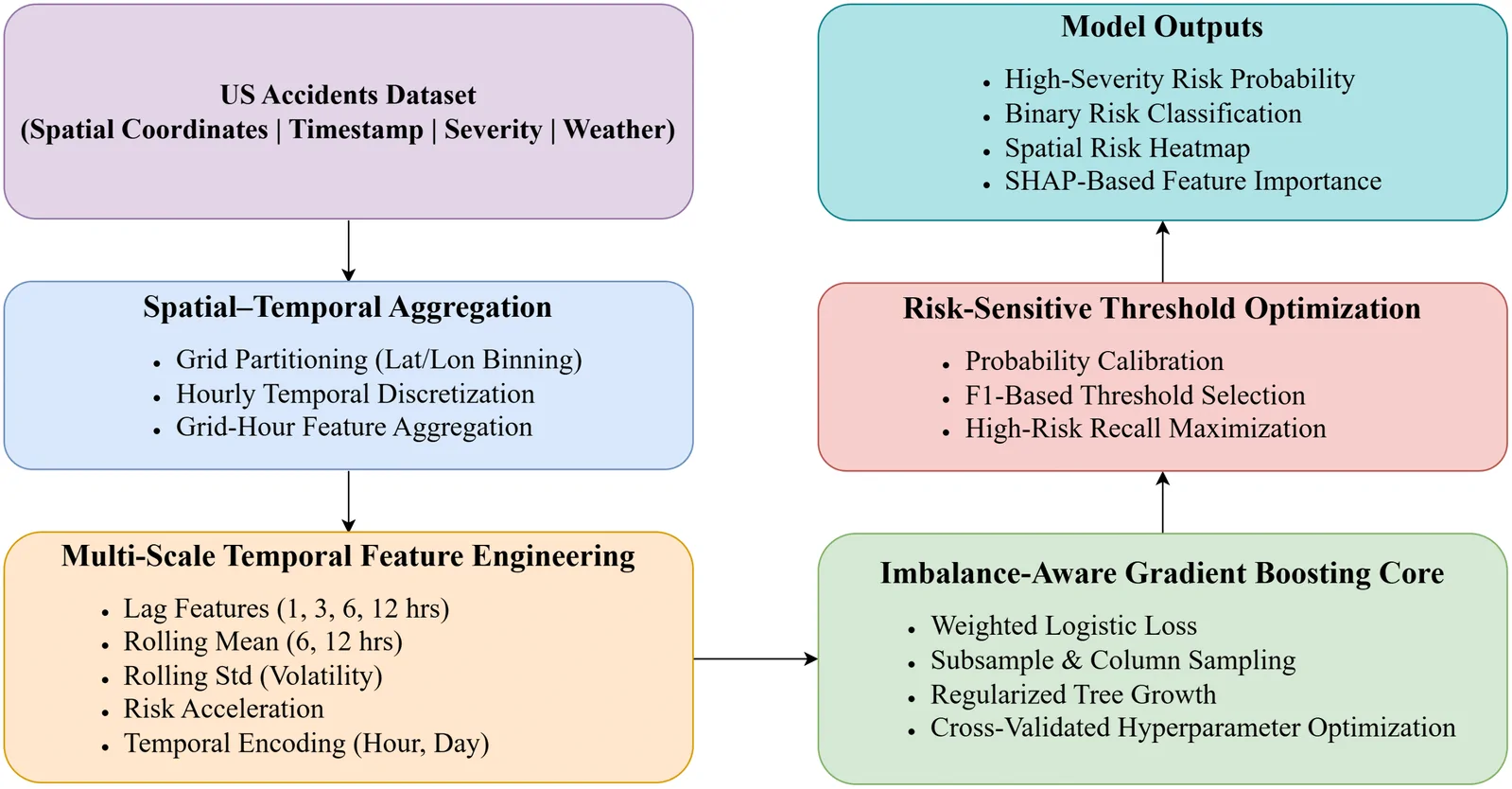
Proposed TRIDENT-Risk architecture for high-severity accident forecasting.

### 4.1. Dataset description

The proposed framework is developed and evaluated using the publicly available US-Accidents dataset((Accessed on: 14/02/2026) https://www.kaggle.com/datasets/sobhanmoosavi/us-accidents/data) [35,36]. This countrywide dataset covers 49 states of the United States and includes accident records collected from February 2016 to March 2023. The data were gathered in real time using multiple traffic APIs that broadcast incident information captured by state departments of transportation, law enforcement agencies, traffic cameras, and road-embedded sensors. The dataset contains approximately 7.7 million accident records and provides rich contextual information including spatial coordinates, timestamps, severity levels, and environmental attributes such as temperature, humidity, and visibility.

For computational feasibility and reproducibility, the dataset is spatially aggregated into regional grids and temporally discretized into hourly intervals. Each grid-time pair represents a spatial–temporal observation used for predictive modeling. High-severity events are defined based on severity levels exceeding a predefined threshold, thereby converting the problem into a binary classification task. The original dataset severity labels were used to define high-severity accidents, which were annotated using the same framework across all states and years in this study. The suggested approach uses standardized severity categories to assure model development and evaluation consistency. This uniform labeling technique reduces geographical or temporal inconsistencies and allows large-scale accident risk prediction. Although minor missing entries may exist due to network disruptions during data collection, appropriate preprocessing and imputation strategies are employed to ensure consistency. To balance spatial granularity, data density, and computational efficiency, the spatial grid resolution was chosen. A coarser grid can mask localized accident patterns due to excessive spatial aggregation, whereas a finer grid may increase spatial resolution but lead to sparse observations and unstable feature estimation. The grid resolution provides enough event density for reliable temporal feature extraction and enough spatial variability for large-scale accident risk prediction.

### 4.2. Spatial-temporal aggregation

Let (Lat, Lon) denote the geographical coordinates of each accident record. The study region is partitioned into uniform spatial grids of size Δ degrees. Each accident is mapped to a grid index *g* using Eq. 14:


$$\begin{array}{c} g=\left(\left\lfloor \frac{\text{Lat}}{\Delta }\right\rfloor ,\left\lfloor \frac{\text{Lon}}{\Delta }\right\rfloor \right) \end{array}$$ (14)

Temporal discretizations is performed at hourly resolution. For each grid *g* and hour *t*, contextual variables are aggregated to form feature vectors ${𝐱}_{g,t}$. These include:

- High-severity accident count
- Total accident count
- Mean temperature
- Mean visibility
- Mean humidity
- Temporal indicators (hour-of-day, day-of-week)

The target variable $y_{g,t}$ is defined in Eq. 15:


$$\begin{array}{c} y_{g,t}=\left\{ \begin{array}{cc} 1, & \text{if at least one high-severity accident occurs within future horizon }H,\\ 0, & \text{otherwise} \end{array}\right. \end{array}$$ (15)

This formulation enables proactive forecasting. Although uniform spatial grids allow computational scalability and reliable aggregation over millions of accident reports, grid-based partitioning may produce the Modifiable Areal Unit Problem (MAUP), where aggregation borders affect spatial patterns. The MAUP may affect prediction performance, but existing research have shown that intermediate grid resolutions often balance geographical detail with statistical stability. Therefore, a fixed grid resolution has been used throughout the study to ensure uniform feature representation and fair model comparison. Combining micro-level roadway factors including intersection layout, lane configuration, curvature, and signal control attributes may smooth them out. This analysis forecasts regional risk, not road-segment crashes. Choose an aggregation technique that balances computational robustness with spatial granularity. Adaptive zoning, graph-based road representations, and road-segment-level learning frameworks will be studied to reduce MAUP impacts.

### 4.3. Multi-scale temporal feature engineering

Temporal dynamics are explicitly modeled using multi-horizon lag and rolling statistics. For each grid *g*, lag features are defined in Eq. 16:


$$\begin{array}{c} {Lag}_{k}(g,t)=H_{g,t-k},\;k\in \{1,3,6,12\} \end{array}$$ (16)

To avoid the leakage of temporal information, all lag features are generated based on the previous accident information seen and available before the prediction period. We define $H_{g,t}$ as the observed high-severity accident count in grid *g* at time interval *t*, which is different from the predicted objec*t*ive.

Rolling mean risk over window *w* is defined in Eq. 17:


$$\begin{array}{c} {\text{RollingMean}}_{w}(g,t)=\frac{1}{w}\sum_{i=1}^{w}y_{g,t-i} \end{array}$$ (17)

Risk volatility is captured using rolling standard deviation as defined in Eq. 18:


$$\begin{array}{c} {\text{RollingStd}}_{w}(g,t)=\sqrt{\frac{1}{w}\sum_{i=1}^{w}{\left(y_{g,t-i}-{\text{RollingMean}}_{w}(g,t)\right)}^{2}} \end{array}$$ (18)

Risk acceleration is defined in Eq. 19:


$$\begin{array}{c} \text{RiskAccel}(g,t)={\text{Lag}}_{1}(g,t)-{\text{Lag}}_{3}(g,t) \end{array}$$ (19)

Consider the lag and rolling properties has been calculated only from previous observations before the prediction instant. The variables ${Lag}_{1}$, ${Lag}_{3}$, ${Lag}_{6}$, ${Lag}_{12}$, rolling mean, rolling standard deviation, and risk acceleration has been derived using historical accident counts ($H_{g,t}$) instead of the future prediction target Independently, the aim variable was at least one high-severity mishap during the one-hour prediction horizon. Thus, the proposed feature engineering technique preserves temporal causality and precludes future observation information leakage into model training.

### 4.4. Imbalance-aware gradient boosting

In this study, class imbalance has been handled exclusively through class-weighted learning using XGBoost’s scale_pos_weight parameter, computed from the training data only. Validation and testing retained the original class distribution. The predictive backbone of proposed TRIDENT-Risk is a gradient boosting ensemble. The model constructs an additive function in Eq. 20:


$$\begin{array}{c} F_{M}(𝐱)=\sum_{m=1}^{M}f_{m}(𝐱) \end{array}$$ (20)

where each $f_{m}$ is a regression tree trained to minimize a differentiable loss. The regularized objective function is defined as Eq. 21:


$$\begin{array}{c} ℒ=\sum_{i=1}^{N}\ell (y_{i},{\hat{y}}_{i})+\sum_{m=1}^{M}\Omega (f_{m}) \end{array}$$ (21)

where ℓ(·) denotes logistic loss and $\Omega (f_{m})$ penalizes tree complexity. To address class imbalance, positive-class weighting is introduced as shown in Eq. 22:


$$\begin{array}{c} \ell (y_{i},{\hat{y}}_{i})=-\alpha y_{i}\log({\hat{p}}_{i})-(1-y_{i})\log(1-{\hat{p}}_{i}) \end{array}$$ (22)

where α is the imbalance weight computed as shown in Eq. 23:


$$\begin{array}{c} \alpha =\frac{N_{\text{negative}}}{N_{\text{positive}}} \end{array}$$ (23)

This weighting enhances sensitivity toward high-risk events.

### 4.5. Risk-sensitive threshold optimization

Instead of using a fixed threshold τ=0.5, TRIDENT-Risk selects $\tau^{*}$ by maximizing the F1-score as defined in Eq. 24:


$$\begin{array}{c} \tau^{*}=\text{arg}\max_{\tau }\left(\frac{2\cdot \text{Precision}(\tau )\cdot \text{Recall}(\tau )}{\text{Precision}(\tau )+\text{Recall}(\tau )}\right) \end{array}$$ (24)

This step ensures balanced detection of high-risk zones while controlling false alarms.

### 4.6. Algorithmic framework

This subsection formally summarizes the computational workflow of the proposed TRIDENT-Risk framework. Each algorithm is accompanied by explanatory context to clarify its role within the overall modeling pipeline.

#### 4.6.1. Algorithm 1: Spatial-temporal aggregation.

The first stage transforms raw accident-level records into structured grid–hour observations. Spatial discretization reduces geographic sparsity and enables regional modeling, while temporal aggregation ensures consistent resolution for forecasting. This step converts event-level data into supervised learning samples.

**Algorithm 1 Spatial-Temporal Aggregation of Accident Data**

**Require:** Raw accident dataset 𝒟 with coordinates and timestamps

**Ensure:** Aggregated grid–hour dataset 𝒢

1: Partition study region into uniform spatial grids of size Δ

2: Discretize timestamps into hourly intervals

3: **for** each accident record in 𝒟 **do**

4:  Map latitude and longitude to grid index *g*

5:  Assign record to corresponding hour *t*

6: **end for**

7: **for** each grid *g* and hour *t* **do**

8: Aggregate contextual features (counts, weather averages)

9:  Define target variable $y_{g,t}$

10: **end for**

11: **return** Aggregated dataset 𝒢

This algorithm ensures consistent spatial–temporal representation and reduces dimensional irregularities inherent in raw streaming data.

#### 4.6.2 Algorithm 2: Multi-scale temporal feature engineering.

Temporal dependencies are explicitly modeled using lag features and rolling statistics. This stage enhances predictive signal by incorporating short-term persistence, medium-term accumulation, and volatility characteristics of accident risk.

**Algorithm 2 Multi-Scale Temporal Feature Construction**

**Require:** Aggregated dataset 𝒢

**Ensure:** Enhanced feature matrix 𝐗

1: **for** each grid *g* **do**

2:  **for** each time step *t* **do**

3:   Compute lag features from historical observed accident counts $H_{g,t-k}$.

4:   Compute rolling mean over windows w∈{6,12}

5:   Compute rolling standard deviation

6:   Compute risk acceleration ${\text{Lag}}_{1}-{\text{Lag}}_{3}$

7:  **end for**

8: **end for**

9: Handle missing values via imputation

10: **return** Feature matrix 𝐗

The engineered variables encode temporal momentum and variability, enabling the ensemble model to capture dynamic risk transitions rather than relying solely on instantaneous observations.

#### 4.6.3. Algorithm 3: TRIDENT-risk training and threshold optimization.

The final stage integrates imbalance-aware gradient boosting with decision-threshold calibration. Weighted optimization ensures sensitivity toward minority high-severity cases, while threshold tuning balances precision and recall.

**Algorithm 3 TRIDENT-Risk Model Training and Optimization**

**Require:** Feature matrix 𝐗, labels 𝐲

**Ensure:** Optimized predictive model $f^{*}$

1:Split dataset into training and testing sets (time-based split)

2: Compute imbalance weight $\alpha =\frac{N_{neg}}{N_{pos}}$

3: Train gradient boosting ensemble with weighted loss

4: Generate predicted probabilities ${\hat{p}}_{g,t}$

5: **for** each threshold τ in candidate set **do**

6:  Compute precision and recall

7:  Evaluate F1-score

8: **end for**

9: Select optimal threshold $\tau^{*}$ maximizing F1

10: **return** Final model $f^{*}$ and threshold $\tau^{*}$

This algorithm operationalization risk-sensitive optimization by explicitly incorporating class imbalance and performance-driven threshold selection. Algorithms 1–3 operate sequentially and collectively define the TRIDENT-Risk pipeline. Spatial aggregation establishes structured regional units, temporal feature construction enhances predictive richness, and imbalance-aware boosting combined with calibrated thresholding produces robust high-risk detection performance. The modular structure also allows scalability to streaming or incremental traffic data scenarios, ensuring adaptability within intelligent transportation systems.

### 4.7. Methodological summary

The TRIDENT-Risk framework integrates large-scale spatial aggregation, multi-scale temporal intelligence, imbalance-aware ensemble learning, and risk-sensitive threshold calibration into a coherent forecasting pipeline. The methodological design ensures robust discrimination, enhanced minority detection, and operational interpretability, making it suitable for proactive safety management within intelligent transportation systems.

## 5. Experimental setup and implementation details

This section describes the data preparation strategy, feature configuration, model implementation details, and evaluation protocol used to ensure reproducibility and fair comparison across baseline and proposed models. Particular attention is given to temporal consistency, imbalance handling, and computational stability, as these factors critically influence accident risk forecasting performance.

### 5.1. Data preprocessing and aggregation

The raw US-Accidents dataset was first filtered to retain relevant attributes including spatial coordinates, timestamp information, severity level, and selected weather variables such as temperature, visibility, and humidity. Records with missing essential spatial or temporal information were removed to maintain structural consistency. Minor missing environmental values were imputed using median-based strategies within spatial–temporal neighborhoods to preserve statistical robustness. Spatial aggregation was performed by partitioning the study region into uniform geographic grids of fixed resolution. Each accident record was mapped to its corresponding grid cell based on latitude and longitude coordinates. Temporal discretizations was performed at hourly resolution, producing grid-hour observations. For each grid and hour, accident counts and contextual averages were computed. This transformation reduced sparsity and enabled structured supervised learning. To preserve the temporal causality, the dataset was ordered chronologically before model construction. The first 80% of observations were used as training data for the models, while the next validation subset was used just for hyperparameter modification and threshold optimization. The last 20% of the observations in chronological order remained back as an unseen test set. No random shuffling was undertaken to avoid predicting past data using future measurements at any time. This approach prevents temporal leakage and better reflects deployment scenarios where future risk must be predicted using historical data. All temporal features were constructed before the prediction horizon using just historical observations, ensuring no future accident information was used. The geographical aggregation resolution was fixed at $\Delta =0.1^{\circ }$ providing a compromise between spatial coverage and data sparsity. The prediction horizon was set to *H* = 1 hour, i.e., in the next hourly interval the framework predicts if at least one high-severity accident will occur or not.

The total dataset had 356,883 grid-hour observations. A chronological train-test split has been used with 285,506 training samples and 71,377 testing samples. Under the adopted evaluation technique, high-severity accident prediction maintained its inherently imbalanced characteristics with a positive-class prevalence of 41.29% in the training set and 25.44% in the testing set as shown in Table 2s Because grid-hour observations may have identical manufactured feature values, repeated feature vectors were found after aggregate during data verification. These distinct spatial and temporal observations were kept for model development and evaluation rather than duplicated accident records.

**Table 2:** Summary of the processed dataset and train-test split.

Dataset Statistic	Value
Processed grid-hour observations	356,883
Training samples	285,506
Testing samples	71,377
Training negative class	167,623 (58.71%)
Training positive class	117,883 (41.29%)
Testing negative class	53,218 (74.56%)
Testing positive class	18,159 (25.44%)

### 5.2. Feature configuration

The final feature space included contextual, temporal, and engineered variables. Contextual variables consisted of accident counts and environmental attributes. Temporal encoding included hour-of-day and day-of-week indicators to capture cyclical mobility patterns. Multi-scale temporal intelligence was incorporated through lag variables at 1, 3, 6, and 12-hour intervals, rolling mean statistics over 6-hour and 12-hour windows, rolling standard deviation to capture volatility, and a risk acceleration term defined as the difference between short-term and medium-term lag values. These engineered features expanded the input dimensionality while preserving interpretability. All numerical features were retained in their natural scale for tree-based models. For neural network baselines, features were standardized using training-set statistics to ensure stable convergence.

### 5.3. Model configuration

All baseline models were implemented using widely adopted ML libraries to ensure comparability. Logistic regression employed $\ell_{2}$ regularization with default solver settings. Random Forest models were configured with 200 trees and balanced class weighting. To ensure fair comparison, hyperparameter optimization was also performed for the primary baseline models using controlled search spaces. The reported results correspond to the best-performing configurations identified on the validation set. The proposed TRIDENT-Risk model utilized an optimized gradient boosting configuration obtained via randomized search with three-fold cross-validation on the training set. The architectural design of proposed model was motivated by the characteristics of large-scale traffic accident data, which exhibit strong class imbalance, heterogeneous feature distributions, nonlinear interactions, and temporal persistence. Conventional deep learning models such as MLPs are highly dependent on balanced training data and often require extensive architectural tuning to effectively learn minority-class patterns. In contrast, gradient boosting ensembles inherently focus on difficult-to-classify samples through iterative residual learning and are particularly effective for structured tabular datasets. The integration of multi-scale temporal features allows the model to capture short-term fluctuations, medium-term accumulation effects, and longer-term risk persistence, while class-weighted optimization increases sensitivity toward rare high-severity events. Finally, threshold calibration aligns the decision boundary with safety-oriented objectives, resulting in improved minority-class recall and PR-AUC performance. The final hyperparameter configuration included a shallow tree depth, moderate subsampling, and controlled learning rate to balance bias and variance. Class imbalance was addressed using a positive class weight computed as the ratio of negative to positive samples in the training set. Validation probabilities were threshold optimized over candidate thresholds from 0.10 to 0.90 in increments of 0.01. The threshold that gave the best validation F1-score was chosen and then applied to the held-out test set. This decreases the possibility of overfitting to the test set, while ensuring that decision boundaries are consistent with operational safety goals. This strategy ensures that the final decision boundary aligns with safety-sensitive detection priorities rather than relying on a fixed probability cutoff.

### 5.4. Hyperparameter configuration

To ensure fair comparison and reproducibility, all baseline models and the proposed TRIDENT-Risk framework were configured using controlled and well-documented hyperparameter settings. For baseline models, standard configurations were adopted to reflect commonly used practical setups, while the proposed model employed hyperparameters selected through randomized search with cross-validation on the training set. The final configuration reflects a balance between generalization capability, computational stability, and minority-class sensitivity. Table 3 summarizes the hyperparameter settings for each model.

**Table 3:** Validated Hyperparameter Configurations of Baseline Models and the Proposed TRIDENT-Risk Framework.

Model	Hyperparameter Configuration
LR	$\ell_{2}$ Regularization; LBFGS Solver
RF	Trees = 200; Max Depth = None; Bootstrap = True; Class Weight = Balanced
XGBoost	Trees = 500; Max Depth = 6; Learning Rate = 0.1; Subsample = 1.0; Colsample = 1.0; Logloss Evaluation
MLP	Hidden Layers = 2; Hidden Units = (64, 32); ReLU Activation; Adam Optimizer
TRIDENT-Risk	Trees = 600; Max Depth = 4; Learning Rate = 0.01; Subsample = 0.7; Colsample = 0.7; Gamma = 0.2; Scale Pos Weight = $\frac{N_{neg}}{N_{pos}}$

#### 5.4.1. Parameter interpretation.

The regularization term in LR constrains the magnitude of coefficients, preventing instability in high-dimensional settings. RF parameters such as the number of trees and bootstrap sampling promote ensemble diversity and reduce variance. The depth parameter controls model expressiveness by limiting recursive partitioning. For gradient boosting models, the number of estimators determines ensemble capacity, while the learning rate governs incremental contribution strength. A smaller learning rate typically enhances stability at the cost of longer training time. Subsample and column sampling ratios introduce stochasticity, improving generalization performance. The γ parameter enforces minimum gain thresholds for tree splitting, reducing overfitting to noise. The imbalance parameter scale_pos_weight plays a crucial role in safety-critical contexts by amplifying the contribution of high-severity samples during loss minimization. This directly improves recall performance without drastically sacrificing discrimination capability. For the multilayer perceptron, the number of hidden units controls representational capacity, while the ReLU activation function ensures nonlinear modeling without vanishing gradients. The Adam optimizer adapts learning rates per parameter to accelerate convergence. To ensure a fair comparative evaluation, the baseline models were configured using validated parameter settings commonly adopted in prior literature and refined through validation-based performance assessment. For tree-based models and neural networks, hyperparameter values were selected after preliminary tuning experiments to obtain stable and competitive performance. The proposed TRIDENT-Risk framework subsequently employed randomized search within a constrained parameter space to further optimize its imbalance-aware forecasting objective. Therefore, performance differences cannot be attributed solely to hyperparameter selection but rather to the integration of temporal feature engineering, imbalance-aware optimization, and threshold calibration. The hyperparameter configuration of TRIDENT-Risk was selected through randomized search with cross-validation to ensure robust performance across temporal splits, thereby preventing overfitting and enhancing operational reliability.

### 5.5. Evaluation protocol

Performance was evaluated using multiple complementary metrics. ROC-AUC was used to assess overall discriminative capability independent of threshold selection. PR-AUC was emphasized due to class imbalance and the importance of minority-class performance. Precision, recall, and F1-score for the high-severity class were reported to quantify operational effectiveness. Accuracy was included for completeness but was not treated as the primary optimization objective. All comparisons were conducted on the identical test set to ensure fairness. Probability-based metrics were computed directly from predicted scores, while classification metrics were derived using the optimized decision threshold.

### 5.6. Implementation environment

The experiments were conducted in a Python-based environment using Scikit-learn for baseline models and XGBoost for gradient boosting implementations. Data manipulation and aggregation were performed using Pandas and NumPy. Spatial visualizations were generated using Matplotlib with high-resolution configuration for publication-quality figures. The experimental pipeline was executed in a cloud-based notebook environment with sufficient memory resources to handle large-scale aggregated datasets efficiently.

The complete workflow—from spatial aggregation and temporal feature engineering to model training and evaluation—was implemented in a modular manner to ensure reproducibility and extensibility. This structured experimental setup enables rigorous comparison between baseline approaches and the proposed TRIDENT-Risk framework, thereby providing a reliable foundation for the subsequent performance analysis.

## 6. Results and discussion

This section presents the quantitative and qualitative evaluation of the proposed TRIDENT-Risk framework. Comparative performance analysis against baseline models is conducted using discrimination, minority-class sensitivity, and operational reliability metrics. Spatial and interpretability analyses are further provided to validate the robustness and practical applicability of the framework.

### 6.1. Quantitative performance comparison

Table 4 summarizes the predictive performance of all evaluated models on the held-out test set.

**Table 4:** Performance Comparison of Baseline Models and Proposed TRIDENT-Risk Framework.

Model	ROC-AUC	PR-AUC	Recall (High-Risk)	F1 (High-Risk)	Accuracy
Logistic Regression	0.8752	0.6941	0.56	0.62	0.83
Random Forest	0.8742	0.6898	0.65	0.65	0.82
XGBoost (Baseline)	0.8770	0.6993	0.64	0.65	0.83
MLP	0.8776	0.7016	0.70	0.67	0.83
**Proposed**	**0.8917**	**0.7266**	**0.81**	**0.69**	0.82

As shown in Table 4, the proposed TRIDENT-Risk framework achieves the highest ROC-AUC of 0.8917, indicating superior discriminative capability across all thresholds. The PR-AUC of 0.7266 further demonstrates improved minority-class precision–recall tradeoff under imbalanced conditions. Most notably, recall for high-severity accidents increases to 0.81, representing a substantial improvement over the strongest baseline (MLP: 0.70). This improvement directly enhances early-warning sensitivity in safety-critical applications. Although overall accuracy remains comparable across models, accuracy is not the primary optimization objective due to class imbalance. The observed increase in minority recall without drastic degradation in precision confirms the effectiveness of imbalance-aware optimization and threshold calibration.

### 6.2. ROC and precision–recall analysis

The ROC curve comparison illustrates consistent dominance of the proposed model across false-positive rates as shown in Fig 2 and The precision–recall curves shown in Fig 3 provide further insight under class imbalance.

**Fig 2:**
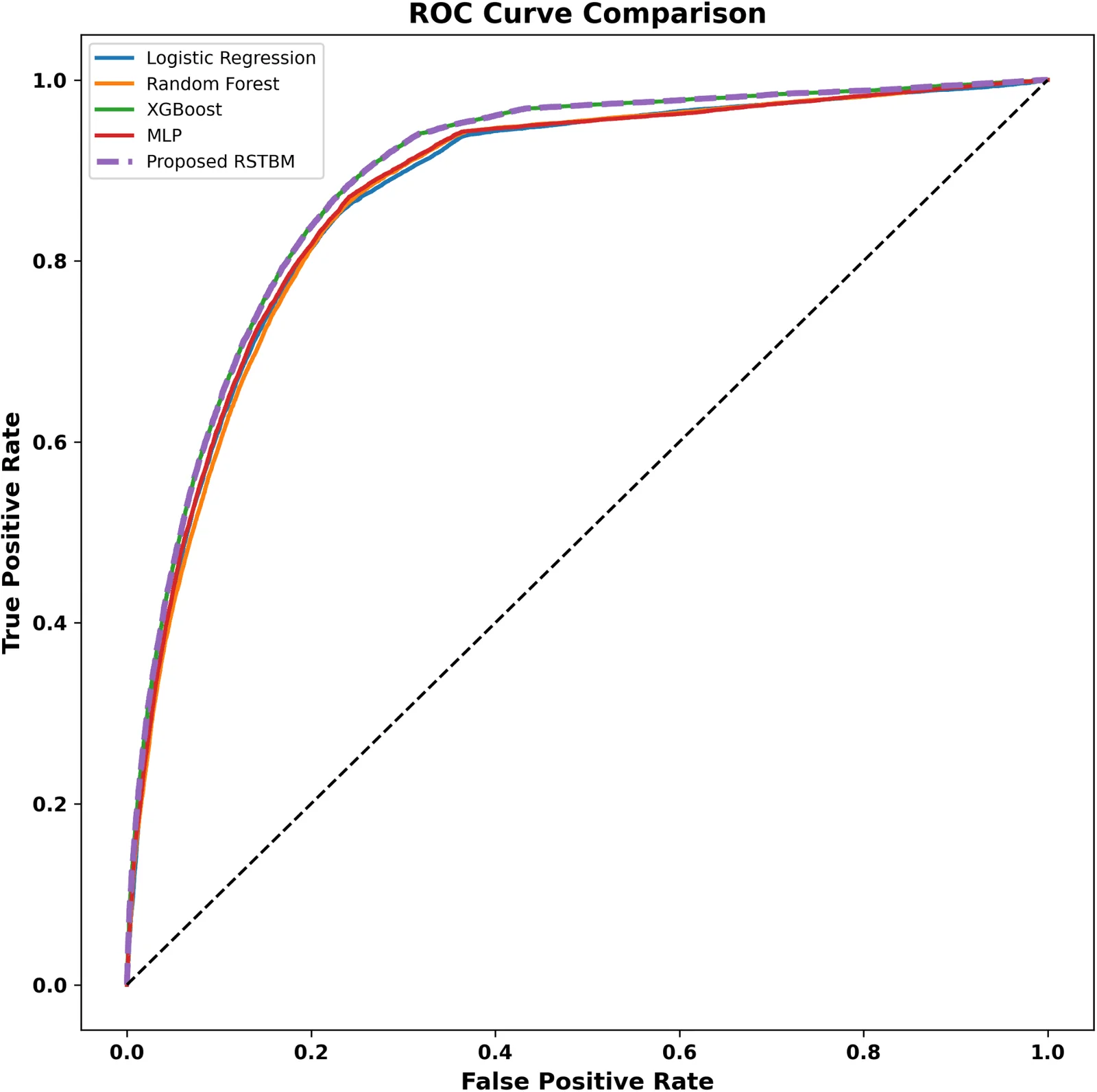
ROC curve comparison of baseline models and the proposed framework.

**Fig 3:**
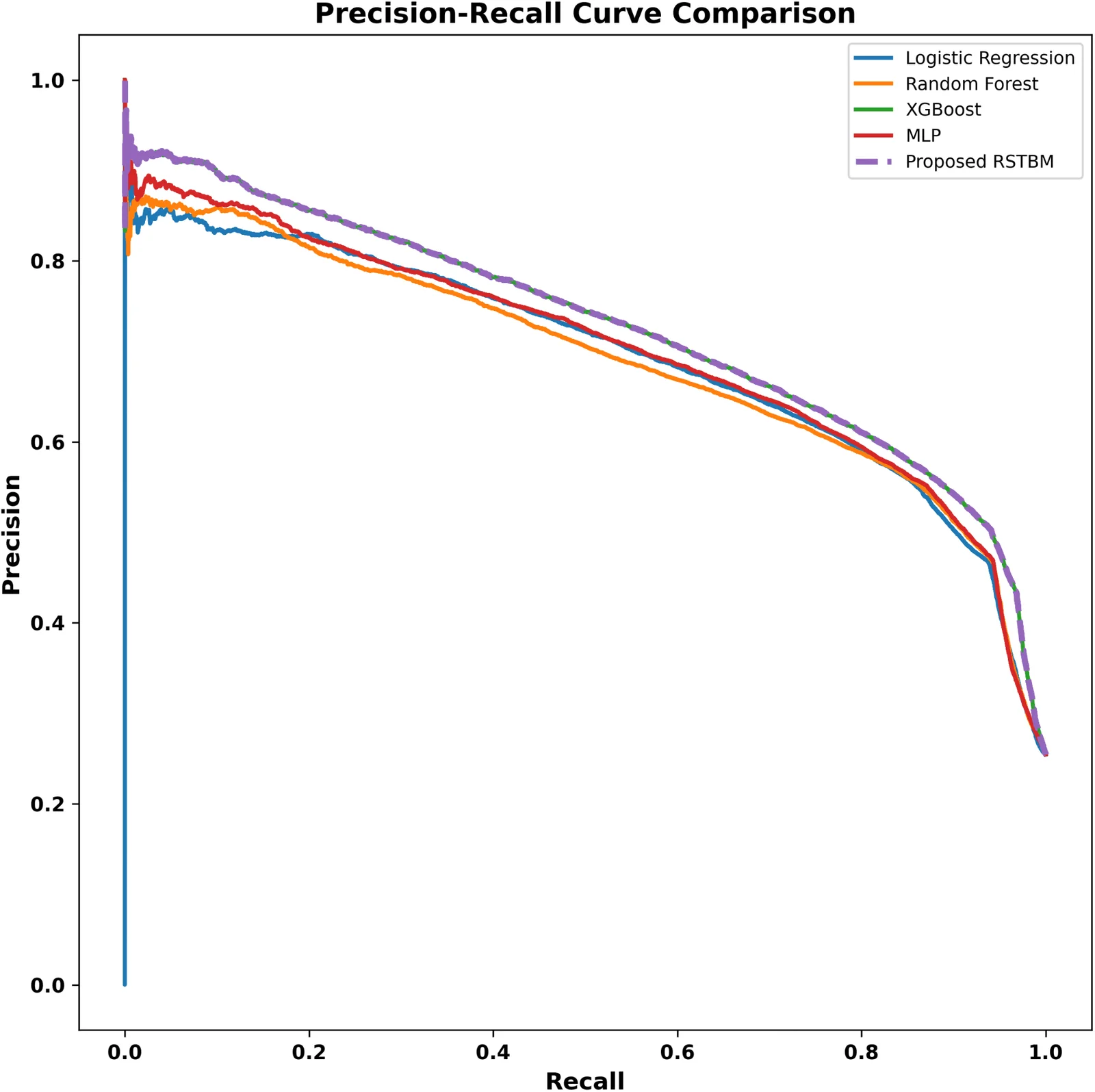
Precision–Recall curve comparison across models.

The proposed framework maintains higher precision across a wide recall range, demonstrating robustness in minority-class detection. The improvement is particularly evident in medium-to-high recall regions, where safety systems typically operate.

### 6.3. High-risk detection performance

Fig 4 compares recall and F1-score for the high-risk class. The substantial increase in recall achieved by TRIDENT-Risk confirms its enhanced capability to detect severe accident conditions. The balanced F1-score further validates that recall gains are not achieved at the expense of severe precision degradation. The multi-metric radar comparison in Fig 5 highlights overall dominance across evaluation criteria.

**Fig 4:**
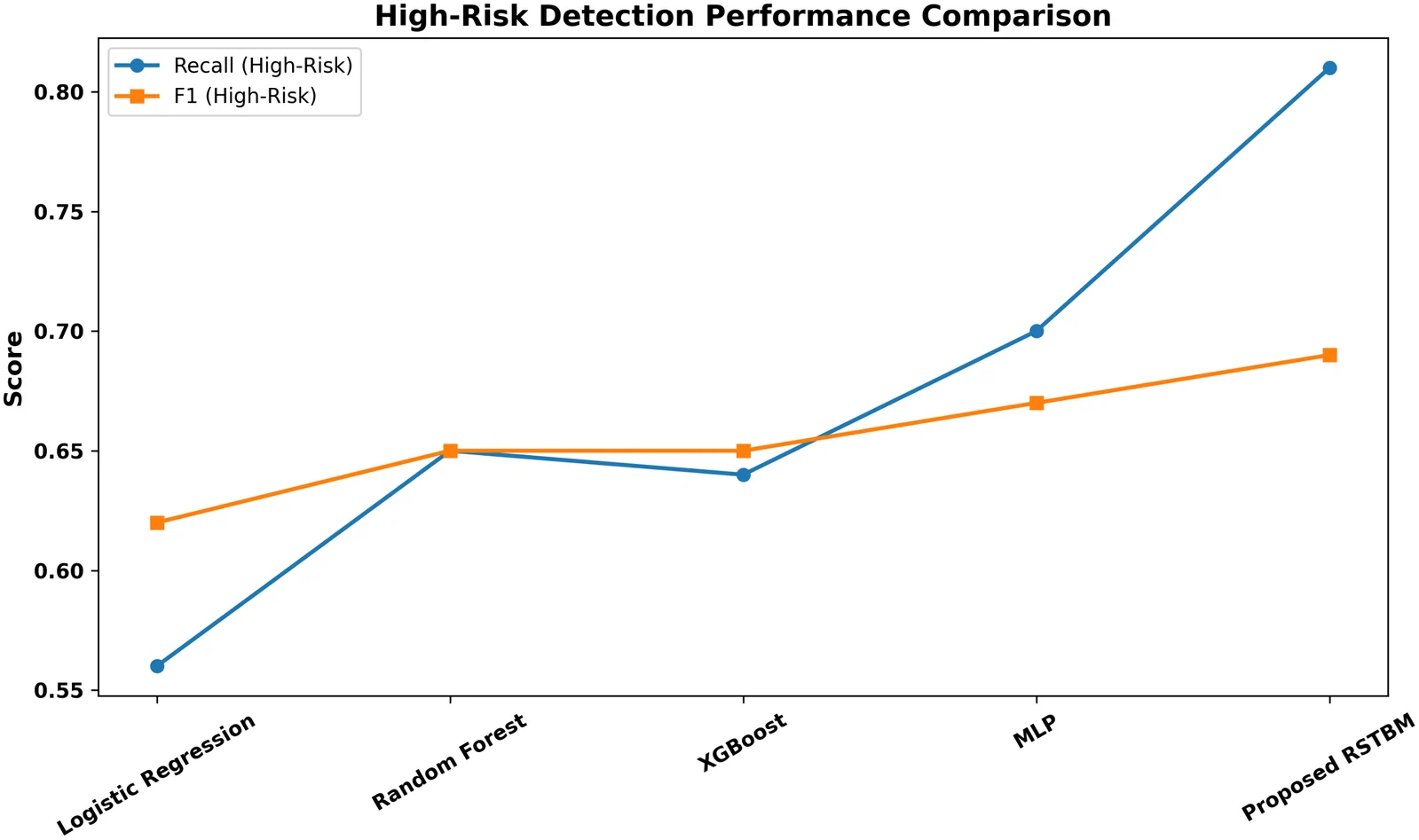
High-risk recall and F1-score comparison across models.

**Fig 5:**
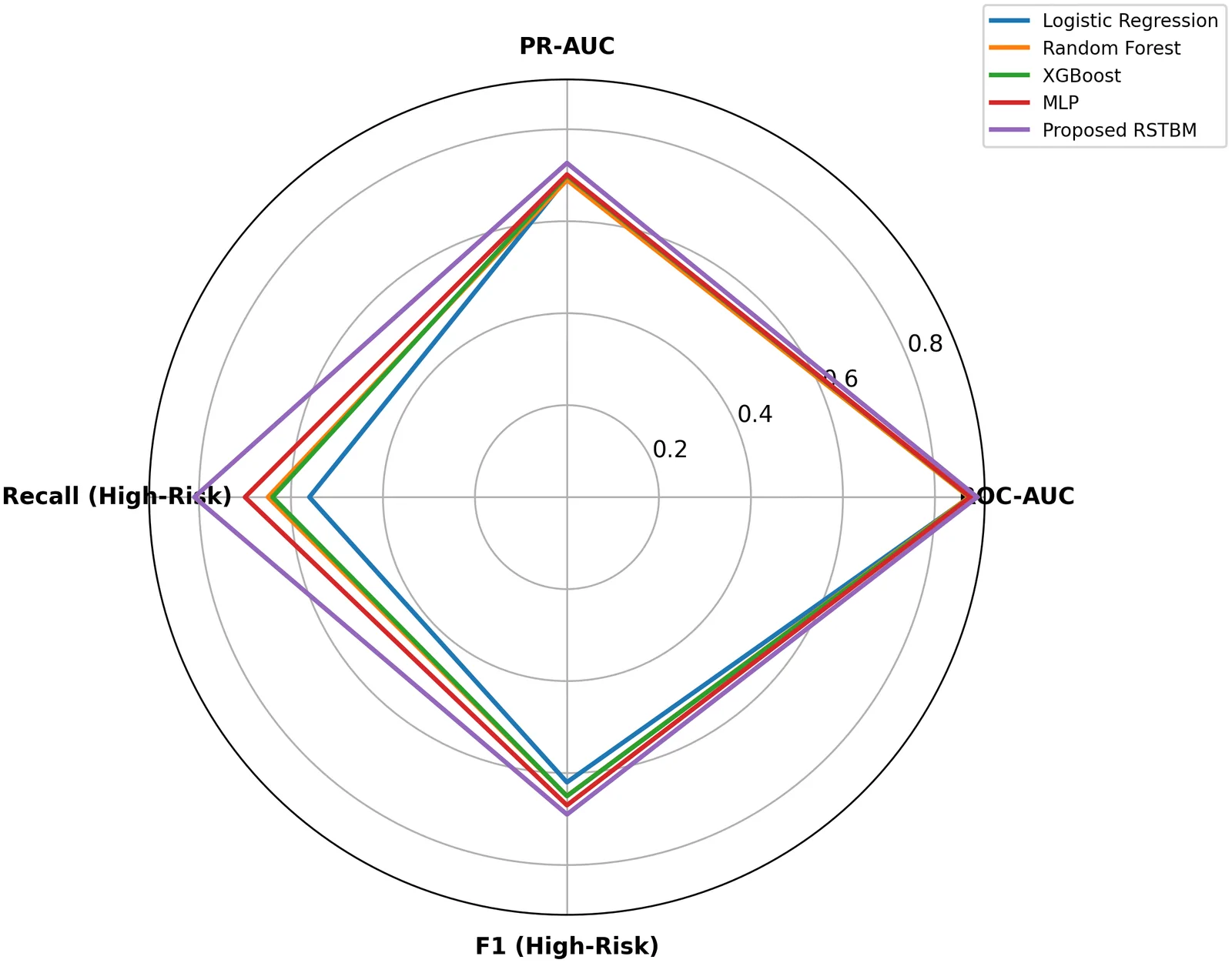
Multi-metric radar comparison of baseline models and the proposed framework.

### 6.4. Spatial risk validation

Spatial validation is illustrated in Fig 6, which overlays predicted risk intensity with actual high-severity accident density.

**Fig 6:**
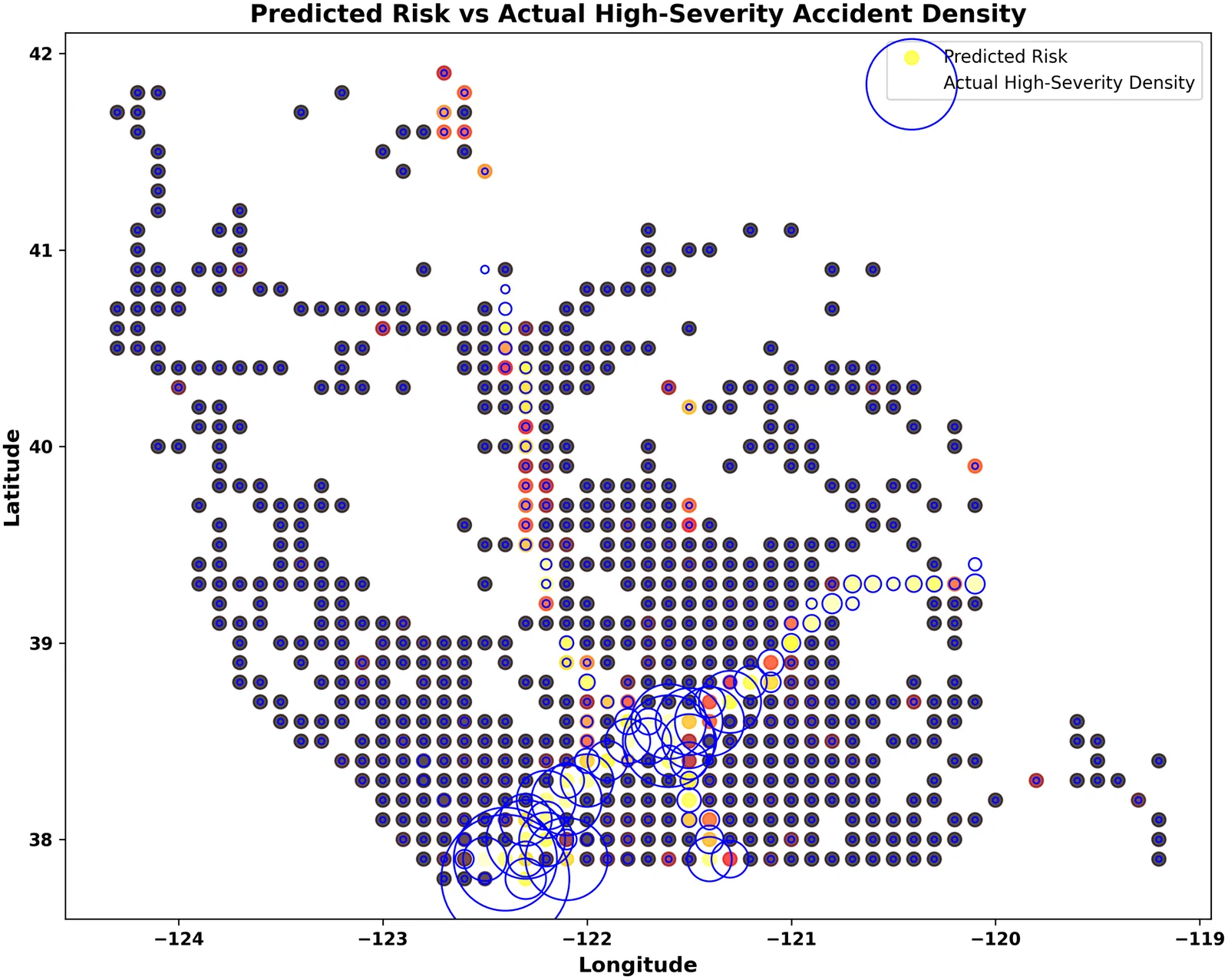
Predicted risk versus actual high-severity accident density.

High-risk zones identified by the model correspond closely with regions exhibiting elevated actual accident density, confirming spatial consistency and practical applicability. This alignment demonstrates that the model captures meaningful geographic patterns rather than overfitting random noise.

### 6.5. Interpretability analysis

To further validate model reliability, SHAP analysis was performed, as illustrated in Fig 7.

**Fig 7:**
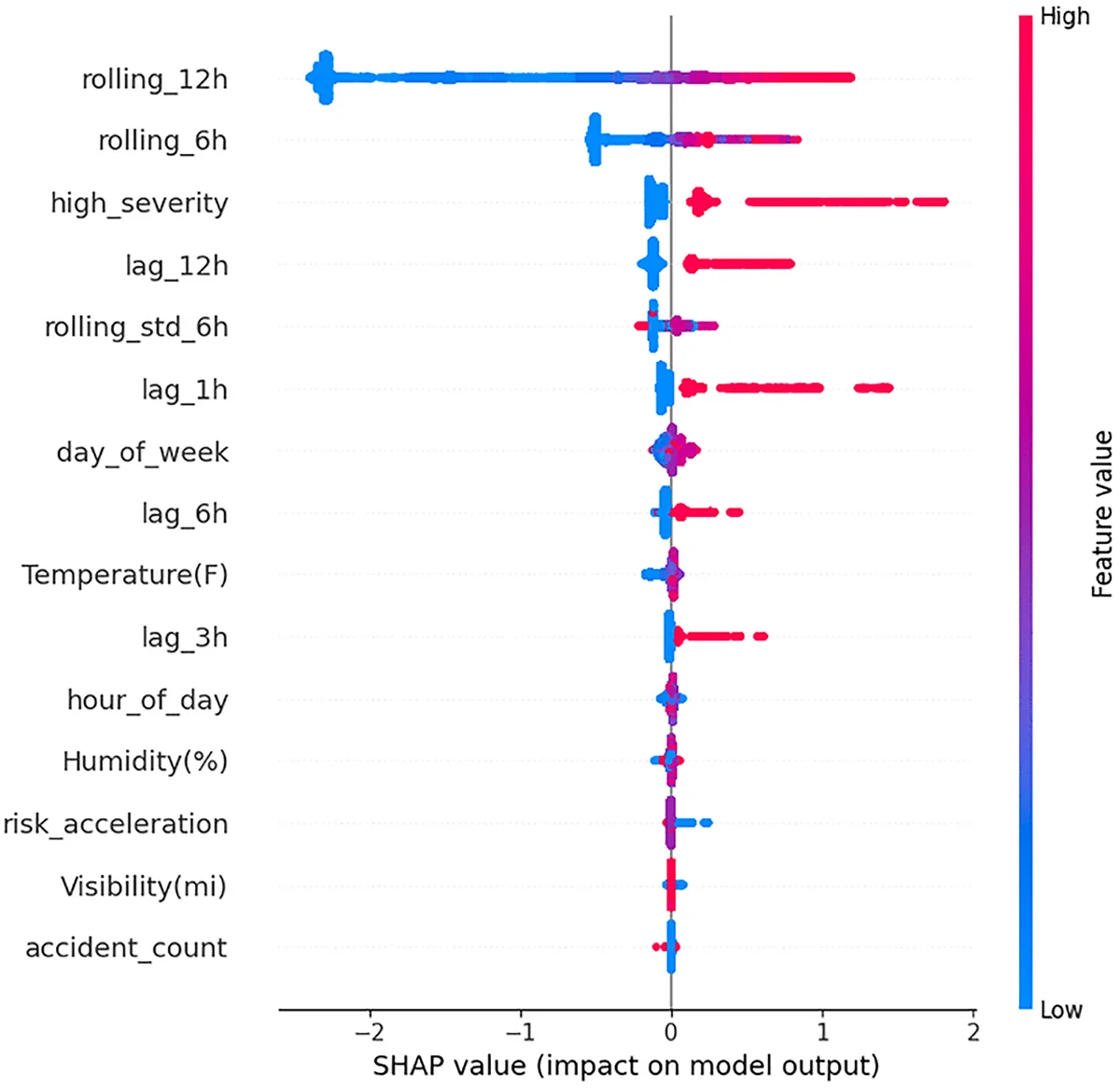
SHAP summary plot illustrating feature importance and impact direction.

The SHAP summary plot indicates that rolling temporal features and lag-based variables contribute most significantly to risk prediction. Environmental attributes such as temperature and visibility exert secondary influence, while static accident count alone provides limited predictive power. This confirms the importance of multi-scale temporal modeling in high-severity accident forecasting. SHAP analysis shows lag and rolling temporal elements as dominant predictors, although the framework is not auto-regressive. The feature space includes environmental parameters, accident density indicators and hour-of-day and day-of-week temporal context descriptors. Timeline train-test separation prevents time leaking and ensures label memorization does not affect expected performance. Accident persistence, not auto regressive bias makes temporal accumulation attributes significant. SHAP values were generated using the TreeExplainer technique, designed for tree-based ensemble models, to increase explainability analysis reliability and consistency. Explanations were based on the independent test dataset, whereas a background sample of 1,000 observations estimated model output. This arrangement ensures consistent and computationally efficient feature attributions, ensuring SHAP values appropriately reflect each feature’s contribution to model predictions.

### 6.6. Statistical validation

To assess the statistical dependability of the proposed TRIDENT-Risk framework, the uncertainty in the model’s predictive performance has been estimated using bootstrap resampling. Specifically, 500 bootstrap iterations were performed by repeatedly sampling the test set with replacement, and the Area Under the Receiver Operating Characteristic Curve (AUROC) was calculated for each iteration. The bootstrap distribution so obtained was used to estimate the mean AUROC and the related 95% confidence interval, which gives a robust assessment of the stability of the model. Furthermore, Receiver Operating Characteristic (ROC) confidence bands were created to test the variability of the classifier at different choice thresholds. Decision Curve Analysis (DCA) was also undertaken to examine the possible clinical and practical usefulness of the proposed framework at different threshold probabilities. The tight confidence intervals and consistent model performance on bootstrap samples show the model is stable and reliable in terms of predictive capabilities, which supports the application of the model for the real-world accident risk prediction.

### 6.7. Calibration analysis

In addition to discrimination performance, the calibration of the proposed TRIDENT-Risk framework was assessed to evaluate the agreement between anticipated probability and the observed occurrence of high-severity incidents. The calibration performance measured using the Brier score, calibration intercept and calibration slope, all measures of the dependability and accuracy of probabilistic predictions. The proposed methodology achieved a Brier score of 0.1594 suggesting an overall strong probabilistic accuracy with reasonably low prediction error. The calibration intercept is −0.2725 with the calibration slope of 1.3470, indicating that the projected probabilities were well aligned with the observed outcomes with small calibration variance. The calibration curve is shown in Fig 8,where the predicted probabilities are close to the ideal calibration line, indicating that the estimated risk is consistent with the actual occurrence of accidents.

**Fig 8:**
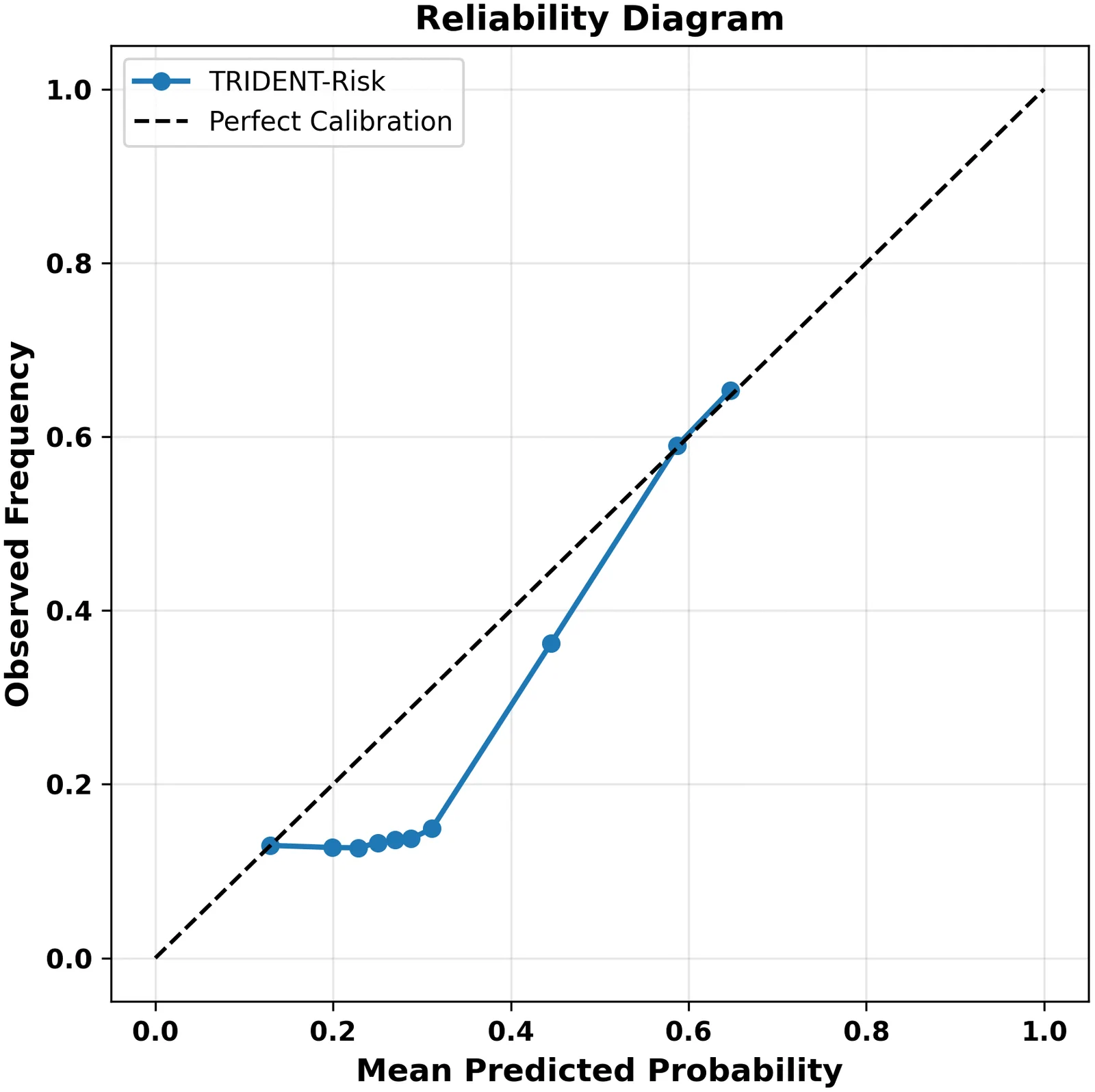
Calibration analysis of the proposed TRIDENT-Risk framework.

The threshold for operational decision-making was 0.5. The precision was 60.66%, and the recall was 57.11%. With this threshold, the proposed architecture generated 17,095 alerts, with an average of 6.69 alerts per day. Of these, 10,370 were real alarms and 6,725 were false alarms with a false alarm rate of 39.34%.

### 6.8. Discussion

#### 6.8.1. Comparative performance analysis.

The experimental results shows that the proposed TRIDENT-Risk framework regularly outperforms baseline models especially in detecting minority-class high-severity events. The proposed TRIDENT-Risk has the highest ROC-AUC of 0.8917 and PR-AUC of 0.7266 outperforming the best baseline MLP at 0.8776 ROC-AUC and 0.7016 PR-AUC. The proposed framework achieved an approximate 15.7% relative improvement in high-risk recall from 0.70 (MLP) to 0.81, while maintaining a competitive F1-score of 0.69 and accuracy of 0.82. The above improvements come from synergistically integrating multi-scale temporal feature engineering, imbalance-aware optimization and adaptive threshold calibration, which lead to more effective learning of temporal risk patterns and significant improvements of minority-class detection for proactive traffic accident forecasting.

#### 6.8.2. Impact of temporal risk features.

One of the main advantage of the proposed framework is that it enables explicit modeling of the temporal evolution of accident risk through multi-scale temporal feature engineering. Instead of depending exclusively on instantaneous observations, the framework employs lag variables, rolling mean statistics, rolling standard deviation and risk acceleration indicators to describe the temporal persistence, volatility and trend dynamics of traffic conditions. Rolling statistics and lag-based variables are the most important predictors which validates the efficacy of the engineering features as per the SHAP analysis. The findings suggest that the occurrence of accidents is substantially correlated with cumulative traffic conditions and not with isolated events. Temporal characteristics allow the model to capture repetitive congestion patterns, long-term bad weather conditions, and progressive risk accumulation that often precedes serious traffic incidents. The integration of temporal intelligence further enhances robustness of the model under highly dynamic traffic situations. The proposed framework can simultaneously capture both short-term fluctuations and medium-term historical dependencies which provides a more comprehensive representation of traffic safety conditions, resulting in better discrimination capability and substantially enhanced detection of high-severity accident events.

#### 6.8.3. Practical implications for intelligent transportation systems.

The proposed TRIDENT-Risk architecture has various practical benefits for intelligent transport systems and traffic safety management. Accurate identification of high-risk areas allows transportation authorities to allocate emergency response resources more efficiently, develop proactive traffic control strategies and provide timely warnings before an accident happens. This ability to foresee can assist in reducing emergency response times and increase overall road safety. The framework is particularly suited to application in modern intelligent transportation infrastructures, since it is based on structured accident and contextual data routinely acquired by traffic monitoring systems, meteorological stations and road-side sensing devices. Furthermore, its modular design allows its smooth integration with current traffic management centers, decision-support systems, as well as smart city infrastructure, without the need for specialized sensor equipment. Another advantage of the suggested framework is the interpretability. The SHAP-based methodology provides straightforward reasons for individual forecasts, which enables transportation engineers and policymakers to identify the primary risk variables affecting the occurrence of accidents. Such transparency boosts users’ faith in the predictive model and aids in evidence-based traffic safety planning, infrastructure upgrading and policy making.

## 7. Limitations and future scope

The proposed TRIDENT-Risk architecture exhibits promising prediction performance, however several limitations still needs to discussed. The system depends on spatial grid aggregation which can smooth out localized road features like intersections and lane patterns. In addition, while a chronologically split train–test split was used to maintain temporal causality, no explicit spatially blocked validation on unseen geographic regions was conducted. Future research directions include extending the framework toward graph-enhanced or hybrid architectures that integrate road network structure with temporal boosting strategies. Incorporating adaptive online learning mechanisms could enable continuous model updating as new accident data become available. Additionally, coupling the predictive model with real-time traffic control systems or decision-support dashboards may facilitate proactive intervention strategies within intelligent transportation ecosystems. Exploring transfer learning across cities or states may further improve scalability and cross-regional adaptability of the framework.

## 8. Conclusion

This study proposed a novel temporal risk-driven and imbalance-optimized ensemble architecture (TRIDENT-Risk) for high-severity traffic accident forecasting. In the proposed system, the spatial-temporal aggregation, multi-scale temporal feature engineering, imbalance-aware gradient boosting and risk-sensitive threshold calibration were combined into a unified predictive pipeline. The proposed model outperformed all the other models including LR, RF, XGBoost and MLP with the greatest ROC-AUC (0.8917) and PR-AUC (0.7266) and significant improvement in recall of minority-class (from 0.70 to 0.81), demonstrating its capability to identify high-risk traffic incidents under extremely imbalanced settings. The experimental results also showed the significant improvement in prediction performance by combining the elements of temporal risk accumulation and imbalance-aware optimization, without increasing the complexity of the model. Spatial validation demonstrated a high consistency between the anticipated risk hotspots and the actual accident distributions. SHAP-based interpretability analysis showed that lag characteristics and rolling risk statistics were the main contributors to the model predictions. The proposed framework offers a scalable, interpretable and practical method to proactively manage traffic safety by enabling early risk assessment, informed decision-making, and data-driven planning in intelligent transportation systems.
